# Assessing rheoencephalography dynamics through analysis of the interactions among brain and cardiac networks during general anesthesia

**DOI:** 10.3389/fnetp.2022.912733

**Published:** 2022-08-29

**Authors:** Carmen González, Gabriel Garcia-Hernando, Erik W. Jensen, Montserrat Vallverdú-Ferrer

**Affiliations:** ^1^ Biomedical Engineering Research Centre, CIBER of Bioengineering, Biomaterials and Nanomedicine (CIBER-BBN), Universitat Politècnica de Catalunya, Barcelona, Spain; ^2^ Research and Development Department, Quantium Medical, Mataró, Spain

**Keywords:** general anesthesia, cerebral blood flow, electroencephalography, rheoencephalography, Poincaré plot descriptors, Granger causality

## Abstract

Cerebral blood flow (CBF) reflects the rate of delivery of arterial blood to the brain. Since no nutrients, oxygen or water can be stored in the cranial cavity due to space and pressure restrictions, a continuous perfusion of the brain is critical for survival. Anesthetic procedures are known to affect cerebral hemodynamics, but CBF is only monitored in critical patients due, among others, to the lack of a continuous and affordable bedside monitor for this purpose. A potential solution through bioelectrical impedance technology, also known as rheoencephalography (REG), is proposed, that could fill the existing gap for a low-cost and effective CBF monitoring tool. The underlying hypothesis is that REG signals carry information on CBF that might be recovered by means of the application of advanced signal processing techniques, allowing to track CBF alterations during anesthetic procedures. The analysis of REG signals was based on geometric features extracted from the time domain in the first place, since this is the standard processing strategy for this type of physiological data. Geometric features were tested to distinguish between different anesthetic depths, and they proved to be capable of tracking cerebral hemodynamic changes during anesthesia. Furthermore, an approach based on Poincaré plot features was proposed, where the reconstructed attractors form REG signals showed significant differences between different anesthetic states. This was a key finding, providing an alternative to standard processing of REG signals and supporting the hypothesis that REG signals do carry CBF information. Furthermore, the analysis of cerebral hemodynamics during anesthetic procedures was performed by means of studying causal relationships between global hemodynamics, cerebral hemodynamics and electroencephalogram (EEG) based-parameters. Interactions were detected during anesthetic drug infusion and patient positioning (Trendelenburg positioning and passive leg raise), providing evidence of the causal coupling between hemodynamics and brain activity. The provided alternative of REG signal processing confirmed the hypothesis that REG signals carry information on CBF. The simplicity of the technology, together with its low cost and easily interpretable outcomes, should provide a new opportunity for REG to reach standard clinical practice. Moreover, causal relationships among the hemodynamic physiological signals and brain activity were assessed, suggesting that the inclusion of REG information in depth of anesthesia monitors could be of valuable use to prevent unwanted CBF alterations during anesthetic procedures.

## 1 Introduction

In the last decades, medical devices have flooded operating theaters to provide healthcare professionals updated and reliable information on patient vital signs, as well as advanced algorithms aiming at improving patient care. Nonetheless, certain clinical signs are not included in standard patient monitoring during surgeries under general anesthesia, such as cerebral blood flow (CBF). Even though CBF is monitored in critical patients, it is not part of the standard of care since it is invasive or extremely unwieldy and expensive.

General anesthetics are known to affect brain hemodynamics, provoking changes in CBF that might interfere in the transit times of the anesthetics towards the target organ, the brain. The main research hypothesis of this research (Slupe and Kirsch, 2018; Tauber et al., 2021; Porta et al., 2022) suggests that CBF plays an important role in anesthesia and might be useful to enhance current algorithms used for depth of anesthesia monitoring. Moreover, to be accepted for standard clinical practice, a CBF monitor to be used for anesthesia titration should be easy to use, non-invasive and cost-effective, provide real time information and guarantee that it does not cause alterations in blood flow during its use.

Rheoencephalography (REG) is an explorative method of cerebral circulation that measures electrical impedance which allows a continuous observation of the blood flow in different cerebral regions. The principle of this method is that blood is a good electrical conductor, therefore any increase in blood volume will lead to a reduction of the brain electrical resistance, and this will be reflected in a decrease of REG pulse amplitude given a constant current. Therefore, REG would comply with the requirements of low-cost and effective CBF monitoring tool (Bodo, 2010; Moskalenko, 2015). REG signals have traditionally been analyzed by assessing the geometrical properties of the blood pulse waves in the time domain (Montgomery et al., 1995; Bodo et al., 2004), such as the duration of the anacrotic phase of the pulse, the maximum and minimum amplitudes, the slope and the area under the curve.

The closest technology to REG is impedance cardiography (ICG), since both share the same working principle based on the electrical bioimpedance. ICG measures the electrical impedance of the thoracic cavity and allows the assessment of several hemodynamic variables, such as cardiac output (CO), stroke volume (SV), left ventricular ejection time (LVET) and systemic vascular resistance (SVR), among others (Siedlecka et al., 2015). Due to the similarities between REG and ICG, and the positive clinical outcome of the use of ICG, the rationale behind the analysis of ICG waves will be applied to REG recordings for CBF estimation.

Within the field of time series nonlinear analysis, many features have been developed for signals characterization, such as the Lyapunov exponents, fractal dimension, Poincaré plot analysis or entropy. Even though none of those algorithms has been applied to REG signals, some authors have studied their performance in processing similar data, such as intracranial pressure (ICP) recordings. For instance (Lu et al., 2012), used multiscale entropy applied to ICP recordings to study their complexity in brain injured patients, concluding that multiscale entropy was a good predictor of mortality and favorable outcome in those patients. Another metric entropy, approximate entropy (ApEn) was selected by (Hornero et al., 2006) to analyze ICP signals in the pediatric population, providing evidence that decreased complexity in ICP was related to events of intracranial hypertension. However, entropy calculations are often cumbersome for real time applications; they could be a powerful tool for post hoc analysis but are not the optimal solution for patient bedside monitoring. In contrast, within the set of nonlinear algorithms applied to biomedical signals, Poincaré plot analysis has shorter computation times and has also been extensively used in physiological signal processing, namely in heart rate variability (HRV) analysis (Khandoker et al., 2013), hence being a suitable tool for REG analysis.

Related to Poincaré plot analysis (Dimitriev et al., 2016), analyzed by means of nonlinear dynamics based on Poincaré plots how the state of anxiety affected heart rate variability. Voss et al. have previously published on the effects of age and gender in short-term heart rate variability analyzed with Poincaré plots among other features (Voss et al., 2015). Other biological signals have been studied by means of Poincaré plots. Hayashi et al. related the delayed coordinates map to changes provoked by anesthesia in the electroencephalogram (EEG) (Hayashi et al., 2015). Hoshi et al. used standard features of Poincaré plot analysis to distinguish between healthy subjects and patients suffering coronary disease, concluding that the SD1/SD2 index provided useful information for that purpose (Hoshi et al., 2013). Even though some features extracted from Poincaré plots are known to be highly correlated to linear time domain information, some others reflect nonlinear behaviors, complementing the diagnosis capabilities of heart rate variability signals, such as the SD1/SD2 parameter or the Complex Correlation Measure (Karmakar et al., 2011).

The causality analysis of different physiological signals has gained popularity in the last decade. Most of the causality studies of biomedical signals have been published on the analyses of causal relationships between heart period, systolic arterial pressure (SAP) and respiration (Faes et al., 2011; Porta et al., 2014). Relevant clinical results have arisen such as the work from (Rield et al., 2010) exploring short-term couplings between respiration, systolic and diastolic blood pressure and heart rate, in order to have a deeper understanding on pre-eclampsia, which is responsible for significant neonatal and maternal mortality. Additionally, Porta et al. studied the causal interactions between heart period, respiration and systolic arterial pressure at rest and after the administration of different drugs, concluding that Granger causality is a suitable tool to describe cardiovascular control and the effects of the administered drugs (Porta et al., 2013). Schulz et al. studied cardiorespiratory causality couplings involved in the processes of an autonomic dysfunction present in patients suffering from schizophrenia (Schulz et al., 2020).

Besides interactions in the hemodynamics system, several publications have focused on the application of Granger Causality on EEG signals. For instance (Juan et al., 2017), studied the connectivity across EEG frequency bands in patients with Alzheimer’s disease, detecting increments of connectivity in the δ band, together with decrement connectivity in other EEG frequency bands. They concluded that Granger Causality (GC) was suitable for Alzheimer diagnosis, since the disconnection among different brain regions is a well-known effect of the disease. Another application of GC in EEG signals was presented by (Coben and Mohammad-Rezazadeh. 2015), who analyzed pre and post-ictal periods of epileptic seizures to study the connectivity between brain regions in epileptic patients.

The GC principles have also been applied to EEG signals during anesthesia. Nicolaou et al., developed a system capable of classifying EEG signals as belonging to awake or anesthetized patients with a 96% accuracy, using as inputs the interactions between EEG signals from different brain areas (Nicolaou and Georgiou, 2014). Moreover, in (Nicolaou et al., 2012) an accuracy of 98% was obtained for loss of consciousness detection, suggesting that GC could be used as an awareness detection system. Barrett et al. analyzed steady state EEG signals during propofol induced anesthesia recorded from the anterior and posterior brain areas, detecting a bilateral increase in GC for the power spectral density in the β and γ bands during loss of consciousness (Barrett et al., 2012).

The interactions between the brain and the hemodynamic system have also been the target of many research projects. Duggento et al. analyzed functional magnetic resonance imaging data, respiration and heartbeat recordings, concluding that GC is a suitable tool to assess causality among brain and heart activity (Duggento et al., 2016). In (Greco et al., 2019), it was studied the causality between hemodynamics and EEG activity during the exposure to pleasant or unpleasant visual stimulation, to relate the reaction to emotions with the changes at the cardiovascular and brain level. Pleasant images increased the coupling from the left hemisphere to the heart, while unpleasant images increased the coupling with the right one, when compared to GC indices at rest. An analysis of brain, cardiovascular and respiratory dynamics was conducted by (Zanetti et al., 2019) combining information-theoretic measures with network physiology during different levels of mental stress. Results indicated that a characterization of these networks is possible in terms of the amount of information transferred within and between the brain and peripheral subnetworks. Faes et al. analyzed causal relationships brain-heart and brain-brain during sleep and concluded that both kinds of interactions were effectively taking place (Faes et al., 2015). Moreover, brain-heart interactions were also studied by (Won et al., 2019) for different sedation levels in anesthetic procedures. EEG spectral power and heart rate signals were analyzed, showing a higher connectivity from brain to heart when compared with the opposite direction for all sedation levels, finding as well a higher coupling in deeper sedation states.

Therefore, the aim of this study is to track cerebral blood flow (CBF) changes during anesthesia by means of rheoencephalography (REG) signals. Thus, REG signals are analyzed using a traditional approach based on the extraction of geometrical properties in the time domain as well as non-linear features extraction by Poincaré plot analysis. Those analyses are applied to different anesthesia scenarios. Moreover, interactions between depth of anesthesia monitoring, REG signal based features and other clinical variables recorded during general anesthesia procedures, such as EEG, infused drugs, heart rate and mean arterial pressure are analyzed by means of causal Granger analysis. This last step aims at detecting cause-effect relationships taking place during general anesthesia procedures, involving interactions between different physiological systems to better characterize the effect of anesthetics on brain hemodynamics.

## 2 Materials and methods

### 2.1 Data acquisition

The analyzed database is composed of 88 female patients enrolled for elective gynecological surgeries under total intravenous anesthesia (TIVA) with propofol and remifentanil. Exclusion criteria considered were cardiac or neurosurgeries, as well as traumatic brain injuries. Summarized demographic data, the set of drugs used for anesthesia management, control of hemodynamics and the occurrence of administration of each drug are specified in Table 1.

**TABLE 1:** General anesthesia dataset.

Patients demographic data	
Age (years)	49.5 ± 16.4
Height (cm)	161.3 ± 7.0
Weight (kg)	68.1 ± 13.9
BMI (kg/m^2^)	26.2 ± 5.2
Drugs administered during surgical procedures	
Propofol	88/88 (100%)
Remifentanil	88/88 (100%)
Rocuronium	43/88 (48.9%)
Atropine	16/88 (18.2%)
Ephedrine	7/88 (7.9%)
Methadone	16/88 (18.2%)

Demographic data values are presented by mean value ± standard deviation.

BMI: body mass index.

The initial dosage of propofol at anesthesia induction was 5.8 μg/ml (ranging from 4.8 to 7 μg/ml) and was administered together with remifentanil targeted at 3.8 ng/ml (ranging from 2 to 6.2 ng/ml). After induction, those target dosages for propofol and remifentanil were reduced to 3.4 μg/ml (from 2.5 to 4.3 μg/ml) and to 3.4 ng/ml (from 2.3 to 4.5 ng/ml), respectively. From the 88 patients suitable for analysis, 22 were intubated through laryngoscopy while in the remaining 66 a Laryngeal Mask Airway (LMA) was used. Patient positioning was also considered in this work, two different positions were assessed besides the standard supine position in steady state anesthesia: 24 patients (27.3%) were kept in the horizontal plane for the whole procedure, while passive leg raising took place in 51 cases (57.9%) and 13 participants (14.8%) were placed in Trendelenburg position (surgical position where the subject lies supine, or flat on their back, with their feet raised higher than their head).

All patients were monitored from 3 min prior to the anesthesia induction until 3 min after extubating. Patient monitoring consisted on the use of a Depth of Anesthesia device, the Conox (Fresenius Kabi, Bad Homburg, Germany) providing the electroencephalogram (EEG) signal and the qCON index that evaluates the hypnotic effects in the brain, as well the qCO (Quantium Medical, Barcelona, Spain) device, an electrical bioimpedance monitor for rheoencephalography (REG) data collection, and a Dräger (Dräger, Lübeck, Germany) hemodynamic monitor for the heart rate (HR in bpm, 1 value/s), systolic blood pressure (SBP, mmHg), diastolic blood pressure (DBP, mmHg) and mean arterial pressure (MAP in mmHg, 1 value/s). Data from the qCO monitor were continuously collected at a sampling frequency of 250 Hz and EEG from Conox with a sampling rate of 1024 Hz, and a resolution of 3 bytes in the range of ± 374 mV.

Data from those monitors, as well as data from the TCI pumps were recorded through the RugloopII software (Demed, Belgium). Moreover, annotation of relevant events during the surgical procedure was performed through the same software, to make sure the occurrence of those events was synchronized with all other clinical data.

The clinical trial followed the principles of the Declaration of Helsinki for human subjects. All participants were informed about the study and gave their written consent prior to participation.

Recorded signals were classified in 5 different categories depending on the clinical state of the patients during general anesthesia:

− Awake, corresponding to the data recorded prior to anesthesia induction.
− Loss of consciousness (LOC), data recorded right after LOC is detected and while intubation is being prepared.
− Steady state anesthesia (Anes), data recorded during anesthesia, without burst suppression episodes (EEG pattern with continuous alternation between high-voltage slow waves or even sharp waves and depressed or even suppressed electrographic activity) and after intubation and patient positioned for surgery.
− Burst suppression rate (BSR), data belonging to periods in which the Conox BSR index provides values higher than 10. The burst suppression rate (BSR), is defined as the fraction of EEG spent in suppression per epoch, is the standard quantitative measure used to characterize burst suppression.
− Recovery of consciousness (ROC), data belonging to the end of the procedure, once drug infusion has been stopped and patient is ready to be extubated.

### 2.2 Signal preprocessing

An automatic artefact rejection algorithm was applied to the recorded EEG signals, in order to avoid processing noisy data resulting from patient movements or the use of other devices, mainly the surgical knife. The traditional frequency band analysis (δ, θ, α, ß) was performed on EEG signals filtered between 0.1 and 50 Hz with a second-order Butterworth filter resampled at 256 Hz. Subsequently, time series were processed in moving time windows of 2 s with 1 s overlap, thus providing new results every second.

REG data were screened for artefact rejection and processed with linear filters. A high-pass filter was applied to REG signals using a fourth-order Chebyshev type II, with 0.1 Hz stop band frequency to eliminate DC fluctuations, followed by a Butterworth second-order low-pass filter with a cut-off frequency at 4 Hz. Subsequent calculations of REG data were applied to sliding windows of 8 s, resulting in a new value every second.

Finally, once time series were preprocessed, they were synchronized with all other data collected during the surgical procedures, such as hemodynamic variables, drug infusion dosages and events recorded during surgery.

#### 2.2.1 Geometric features of rheoencephalography signals

The classical methods used to assess REG signals rely on the analysis of the geometry of the pulse waves (González et al., 2018). In this way, for REG recordings, the minimum and maximum values of each pulse wave and their respective derivatives were automatically detected, and the following features were calculated: amplitude range of the pulse (Range), time between two consecutive maximums (Δtmax, samples), time between two consecutive minimums (Δtmin, samples), time between each minimum and the following maximum (Δtmin-max, samples), the slope (α, a.u.) of the pulse during Δtmin-max interval, the area under the pulse wave (Area, *Ω* s), the systolic area (AreaSyst, *Ω* s) that is calculated as the area of a pulse wave delimited by a minimum and its consecutive maximum, the maximum derivative (δmax, *Ω* s^−1^) and the range of the derivative (δrange, *Ω* s^−1^). In addition, blood volume and blood flow estimations were also considered. The relative cerebral blood volume (CBVrel, Ω) was calculated as:

CBVrel=δmaxLVET (1)

where the left ventricular ejection time (LVET, ms) was considered as a function of HR_REG_ (bpm), LVET = 416–1.56 HR_REG_ (Willems et al., 1970), computing HR_REG_ from the difference between two consecutive maximums of the REG curve. The cerebral blood estimation (CBFest, *Ω* s^−1^) was calculated as:

$$\mathrm{CB}\mathrm{Fest}=\mathrm{CBVrel}{\mathrm{HR}}_{REG}/60$$ (2)

#### 2.2.2 Poincaré plot analysis of rheoencephalography signals

**FIGURE 1:**
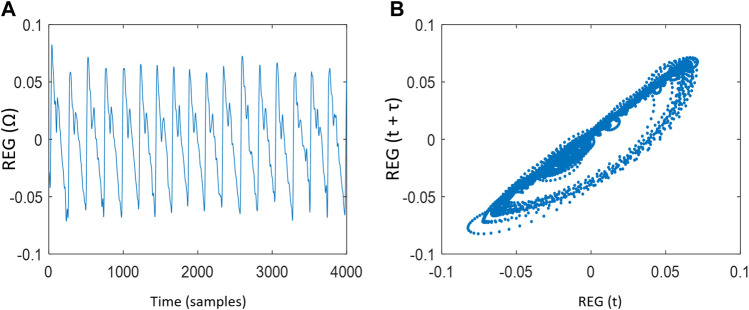
**(A)** Rheoencephalography (REG) signal trend and **(B)** Poincaré plot reconstruction of a REG signal with time lag τ = 5 samples.

Two-dimensional Poincaré plot was constructed from REG sequences, with REG(t) at *x*-axis and REG (t+τ) at *y*-axis, where t moves from 1 to N-τ samples, being N the length of the series. The choice of the time lag *τ* is critical, since very low values would not allow the attractor to expand, with a majority of points laying on the diagonal line (Chen at al., 2007), while very large values of *τ* would cause deformations of the attractor due to the fact that pairs of samples would be uncorrelated (Fraser and Swinney, 1986; King et al., 1987). Since no previous work has been done on the analysis of REG attractors during general anesthesia, a wide range of *τ* values was analyzed (from 1 to 20 samples) in order to provide a *τ* value able to give the maximum possible information related to the dynamics hidden in REG signals. Figure 1 shows a rheoencephalography (REG) signal trend and its related Poincaré plot reconstruction of a patient.

To generate quantitative information on the distribution of REG signals in the Poincaré plots, several features were extracted from the reconstructed attractor:

− SD1 and SD2 which respectively are the standard deviation (SD) of REG(t) dispersion perpendicular to the diagonal line (the identity line) and the SD of the REG(t) dispersion along the diagonal line. They are computed following Eq. 3 where var is the variance.

$$\text{SD}1=\sqrt{\text{var}\left(\frac{\text{REG}\left(\text{t}\right)-\text{REG}\left(\text{t}+\text{τ}\right)}{\sqrt{2}}\right)}\;\text{SD}2=\sqrt{\text{var}\left(\frac{\text{REG}\left(\text{t}\right)+\text{REG}\left(\text{t}+\text{τ}\right)}{\sqrt{2}}\right)}$$ (3)

− Area of the ellipse (SDarea), calculated as SDarea = *π* SD1 SD2
− Ratio SDratio, defined as SD1/SD2
− Correlation R, measured between REG(t) and REG (t+τ) signals.
− Complex correlation measure (CCM) (Karmakar et al., 2009), identifies all possible sets of three consecutive attractor points of the Poincaré plot and the area of the triangle they define is calculated (González, et al., 2018). In cases where all three points are aligned, the area is considered to be zero. CCM is computed as indicated in Eq. 4:

$$\text{CCM}\left(\text{τ}\right)=\frac{1}{\text{SDarea }\left(\text{N}-2\right)}\sum_{\text{i}=1}^{\text{N}-2}\left\|\text{M}\left(\text{i}\right)\right\|$$ (4)

where M(i) is the matrix including the coordinates of the three points from each subset whose determinant is the area of the triangle formed by them and SDarea is the normalizing constant and represents the area of the fitted ellipse over Poincaré plot.

#### 2.2.3 Granger causality analysis

Granger Causality (GC) has been applied to the collected signals to assess causality between pairs of time series. It relies on a hypothesis test in which the null hypothesis is that, given two time series x_1_(t) and x_2_(t), x_2_(t) does not cause x_1_(t). In order to assess the causality between the signals, autoregressive models (AR) are built, the restricted and the unrestricted model. The restricted model (univariate AR model) uses only past values from the signal x_1_(t) to predict its future values, while the unrestricted model (bivariate ARX model) uses past values from both x_1_(t) and x_2_(t) to predict values of x_1_(t). The restricted model is defined as:

$${\text{x}}_{1}\left(\text{t}\right)={\text{a}}_{1}+\sum_{\text{i}=1}^{\text{L}}{\text{a}}_{1,\text{i}}{\text{ x}}_{1}\left(\text{t}-\text{i}\right)+{\text{ ε}}_{1\text{r}}\left(\text{t}\right)$$ (5)

$${\text{x}}_{2}\left(\text{t}\right)={\text{a}}_{2}+\sum_{\text{i}=1}^{\text{L}}{\text{a}}_{2,\text{i}}{\text{ x}}_{2}\left(\text{t}-\text{i}\right)+{\text{ ε}}_{2\text{r}}\left(\text{t}\right)$$ (6)

while the unrestricted model are represented by:

$${\text{x}}_{1}\left(\text{t}\right)={\text{b}}_{1}+\sum_{\text{i}=1}^{\text{L}}{\text{b}}_{1,\text{i}}{\text{ x}}_{1}\left(\text{t}-\text{i}\right)+\;\sum_{\text{i}=1}^{\text{L}}{\text{c}}_{1,\text{i}}{\text{ x}}_{2}\left(\text{t}-\text{i}\right)+{\text{ ε}}_{1\text{u}}\left(\text{t}\right)$$ (7)

$${\text{x}}_{2}\left(\text{t}\right)={\text{b}}_{2}+\sum_{\text{i}=1}^{\text{L}}{\text{b}}_{2,\text{i}}{\text{ x}}_{2}\left(\text{t}-\text{i}\right)+\;\sum_{\text{i}=1}^{\text{L}}{\text{c}}_{2,\text{i}}{\text{ x}}_{1}\left(\text{t}-\text{i}\right)+{\text{ ε}}_{2\text{u}}\left(\text{t}\right)$$ (8)

where a_ji_, b_ji_ and c_ji_ are the estimated coefficients of the models of order L, being j = {1,2} and the residuals (prediction errors) of the models are ${\text{ε}}_{\text{jr}}\left(\text{t}\right)$ and ${\text{ε}}_{\text{ju}}\left(\text{t}\right)$ .

The Schwartz’s Bayesian Information Criterion (BIC) (Schwartz 1978) was selected to fit the order L of the model, since it has been published to be more consistent (Zhang, 1993) and demonstrated (Nicolau and Georgiou, 2013) to provide reliable values for EEG models under general anesthesia. The optimal order L was *a priori* tested from 1 to 10 samples (i.e. 10 s).

To decide if the null hypothesis is rejected, an analysis of variance test was carried out. In this context, F-statistic is computed as:

$$\text{F}=\frac{\text{SSR}/{\text{d}}_{\text{n}}}{\text{SSE}/{\text{d}}_{\text{d}}}$$ (9)

where SSR is the sum of squares explained by the regression, SSE is the sum of squares errors, d_n_ equals the number of independent variables and the degrees of freedom of the SSE are d_d_ = N - d_n_ -1. If the statistic is found significant at level *p*-value<0.05, the null hypothesis is rejected and causality from the time series x_2_(t) to x_1_(t) is considered to take place. Following a similar procedure, the causality from times series x_1_(t) to x_2_(t) is evaluated. The magnitude of the causality from x_1_(t) to x_2_(t) and x_2_(t) to x_1_(t) was measured respectively as function of the model error variances:

$$\begin{array}{c} {\text{C}}_{x1\to x2}=\text{ln }\frac{\text{var}\left({ɛ}_{2\text{r}}\right)}{\text{var}\left({ɛ}_{2\text{u}}\right)}\\ {\text{C}}_{x2\to x1}=\text{ln }\frac{\text{var}\left({ɛ}_{1\text{r}}\right)}{\text{var}\left({ɛ}_{1\text{u}}\right)} \end{array}$$ (10)

#### 2.2.4 Data analysis and statistical analysis

The features extracted from each constructed two-dimensional Poincaré plot on REG(t) signals were SD1, SD2, SDratio, SDarea, R and CCM. A statistical analysis was performed to select the *τ* value from 1 to 20 samples (based on González et al., 2018) that allows those features to statistically distinguish the clinical states of a general anesthesia (Awake, LOC, Anes, BSR, ROC).

Furthermore, a statistical analysis was applied on REG geometric features (Range, Δtmax, Δtmin, Δtmin-max, Slope α, Area, AreaSyst, δmax, δrange, CBVrel and CBFest), REG Poincaré plot descriptors (SD1, SD2, SDratio, SDarea, CCM and R), global hemodynamics (HR, MAP), the effect site concentrations of propofol and remifentanil (CePropo, CeRemi) and EEG based-parameters related to depth of anesthesia (qCON and the EEGδ, EEGθ, EEGα and EEGß energy bands) in order to statistically study their performance in discriminating between the consecutive clinical states of the patients during general anesthesia.

For each patient, all these descriptors were calculated on each time-varying 8-s sliding-window at each state and their averaged value calculated. ANOVA for repeated measures for normal distributions and Friedman test for non-normal distributions verified by the Kolmogorov–Smirnov test were applied. This analysis was followed by the post hoc non-parametric paired samples Wilcoxon test. A significant level *p*-value<0.01 (Bonferroni correction) was considered.

Causality analysis was applied among pairs of simultaneous feature values, calculated on each time-varying 8-s sliding-window obtained from each patient undergoing general anesthesia. All windows of all data were synchronized. The features taken into account were those REG(t) geometric descriptors (named as CBF lin), REG(t) Poincaré plot descriptors (named as CBF PP), global hemodynamics features (HR and MAP), the effect site concentrations of propofol (CePropo) and remifentanil (CeRemi) and EEG based-parameters. The coupling strength between those families of features were analyzed under different general anesthesia events:

a) Steady state anesthesia (n = 84 segments): 400s periods in which effect site concentrations of propofol and remifentanil were constant, and no surgical events took place.
b) Propofol infusion (n = 29 segments): periods from 200s before to 200s after the change of the target effect site concentration of propofol, while remifentanil was kept constant.
c) Remifentanil infusion (n = 16 segments) periods from 200s before to 200s after the change of the target effect site concentration of remifentanil, while propofol was kept constant.
d) Atropine infusion (n = 16 segments): periods from 200s before to 200s after the administration of atropine.
e) Ephedrine infusion (n = 7 segments): periods from 200s before to 200s after the administration of ephedrine.
f) Trendelenburg position (n = 12 segments): periods from 200s before to 200s after the positioning of the patient from the horizontal supine position to the Trendelenburg position.
g) Passive leg raising (n = 48 segments) periods from 200s before to 200s after the elevation of patient legs in preparation for surgery.

Among the set of clinical events in which causality was studied, the periods of steady state anesthesia were used as reference, thus the results from the other events, such as atropine infusion or Threndelenburg positioning were compared to those obtained during stable anesthesia.

Given a pair of variables x_1_(t) and x_2_(t), causality indices ${\text{C}}_{x1\to x2}$ and ${\text{C}}_{x2\to x1}$ were compared through statistical hypothesis testing. Normality of the data was assessed by means of a Kolmogorov-Smirnov test and subsequently, t-student test was applied. This analysis was followed by the post hoc non-parametric U Mann-Whitney test. Statistical significance level *p*-value < 0.005 was considered.

Causality diagrams are drawn for each general anesthesia event. Whenever causality indices were higher in one-way, with statistical significance, this direction of causality is considered and represented in the causality diagrams with a single arrow. The occurrence of the interactions between two groups was computed as the number of patients presenting at least one statistically significant causal relationship between any pair of features belonging to the two groups under analysis.

Moreover, for each event, Spearman correlations (ρ) between the causality indices and patient demographics were calculated and considered as confounding factors for *p*-value <0.01, due to the large number of correlations being analyzed simultaneously. Only correlations reaching absolute values above 0.5 were included for analysis. Those relations that presented a correlation higher than 0.5, a regression analysis based on one variable was constructed for analyzing the influence of patient demographics on causality indices.

## 3 Results

### 3.1 Estimating Poincaré plot time-lag on rheoencephalography signals

To determine the time lag *τ* of the Poincaré plot of REG sequences able to provide the maximum possible information related to the dynamics hidden in REG signals, a wide range of *τ* values is studied (*τ* = {1, …, 20} samples).

**FIGURE 2:**
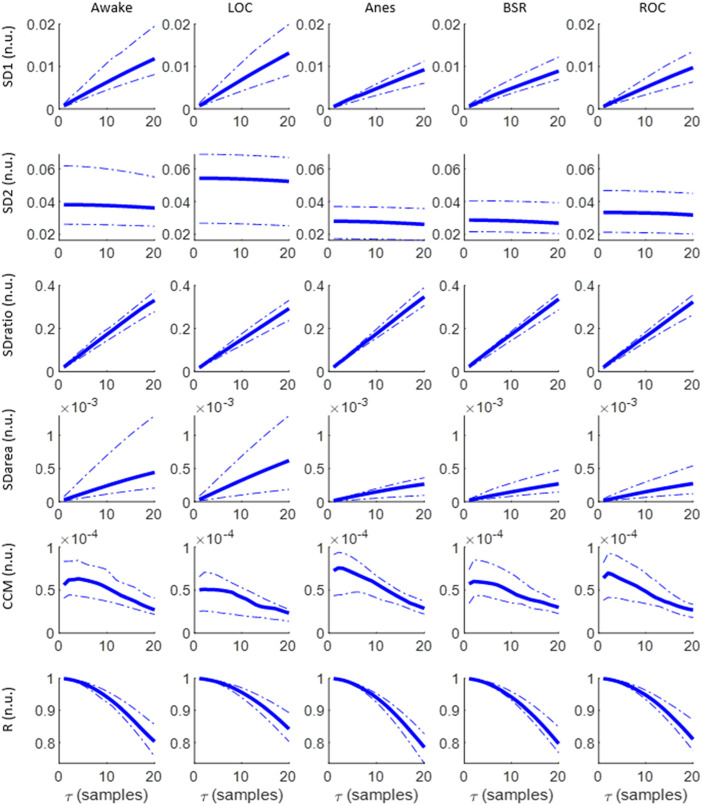
Evolution of SD1, SD2, SDratio, SDarea, CCM and R as a function of τ for the set of anesthesia states under analysis: Awake, LOC, Anes, BSR and LOC. Median values are graphed, together with the 25th and 75th quartiles represented with dashed lines.

The evolution of each Poincaré plot descriptor as a function of the time lag *τ* for each anesthesia phase is depicted in Figure 2. SD1 increases as *τ* increases in all states, reaching higher values for awake and LOC, which are also characterized by a wider interquartile range. In contrast, SD2 remains stable for all *τ* values, providing a higher score during Awake and LOC states. Subsequently, their ratio (SDratio) increases as *τ* increases, with similar interquartile ranges among the various anesthesia stages, while the ellipse area (SDarea) shows higher values for Awake and LOC, with a higher dispersion in those two states as *τ* increases compared with dispersions of Anes, BSR and ROC states. The behavior of the correlation R decreases for increasing *τ* values in all anesthesia phases, and showing similar interquartile ranges across states. Finally, CCM is the only feature showing a local maximum, identified in low *τ* values (*τ* ≤ 5) and providing its highest values in Anes state.

**FIGURE 3:**
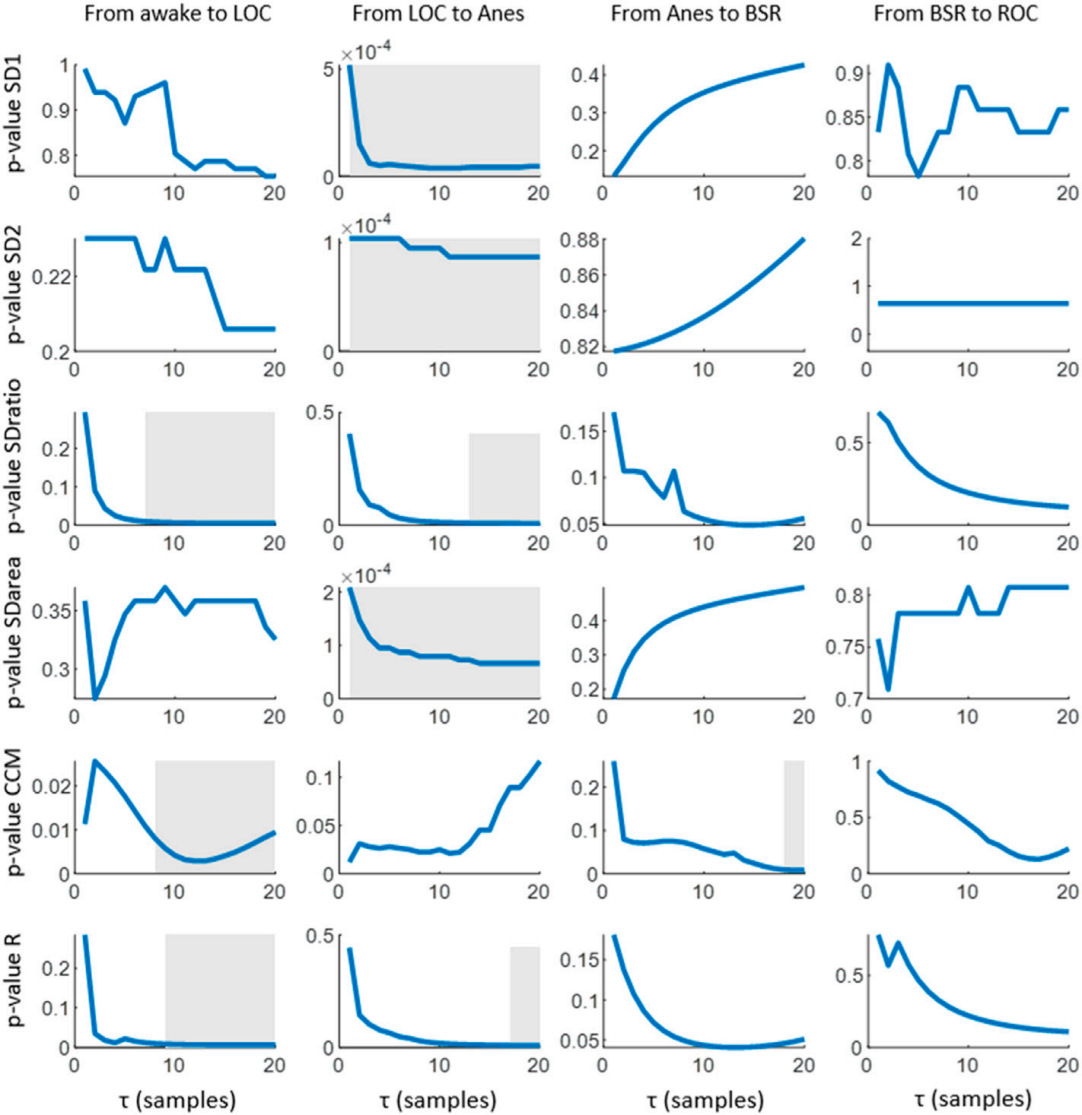
Statistical significance (*p*-values) obtained for the comparison of the median values of each Poincaré feature (SD1, SD2, SDratio, SDarea, CCM and R) among consecutive anesthesia states. The post hoc non-parametric paired samples Wilcoxon test was applied. Grey areas represent intervals in which the graphed parameter shows statistical significance of *p*-value<0.01.

All the extracted features (SD1, SD2, SDratio, SDarea, CCM and R) present differences between the targeted set of anesthetic states. The statistical significance of those differences is assessed in Figure 3, SD1 and SD2 showed the ability to differentiate between LOC and Anes (*p*-value <0.01) for all *τ* values, while they failed in reflecting differences among all other transitions between consecutive states. Regarding SDratio, significant differences were detected in both transitions Awake vs LOC and LOC vs Anes. Nonetheless, the *τ* range in which *p*-values were under the significance threshold (*p*-value <0.01) was reduced to the intervals 8 to 20 samples and 12 to 20 samples, respectively.

The correlation R provided a similar performance but with narrower *τ* ranges for significance: 10 to 20 samples for the transition between Awake and LOC and 17 to 20 samples for LOC and Anes. The ability of SDarea to distinguish between consecutive states was limited to the transition between LOC and Anes, preserving the statistical significance for all *τ* values tested. CCM is the only feature that does not distinguish between LOC and Anes, but it provides statistical significant results (*p*-value <0.01) in the transition between Anes and BSR for *τ* from 18 to 20 samples. Moreover, it also reflects differences between Awake and LOC states for *τ* > 10 samples.

Considering the selection of the optimal *τ* values to assess differences between consecutive anesthesia states (Awake vs LOC, LOC vs Anes, Anes vs BSR and BSR vs ROC) using the set of features extracted from the Poincaré plot, low *τ* values have proved to fail in reflecting changes while the highest range of the tested interval showed a better performance considering all features and anesthesia states (Figure 3)**.** Therefore, the value *τ* = 20 samples was chosen to be appropriate to detect changes in anesthesia states.

### 3.2 Analysis of the behaviors of the anesthesia state descriptors

**FIGURE 4:**
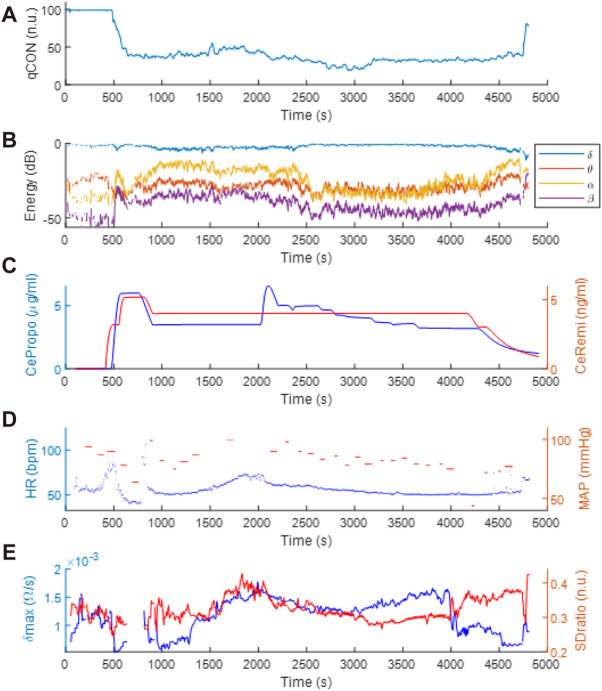
Clinical data recorded during anesthetic procedure: **(A)** qCON index, **(B)** EEG frequency bands, **(C)** propofol and remifentanil effect site concentrations (CePropo and CeRemi, respectively), **(D)** heart rate (HR) and mean arterial pressure (MAP) and **(E)** δmax and SDratio REG features.

Figure 4 includes a set of data recorded from one subject participating in the clinical trial. The anesthesia induction started at t = 500s approximately, with the infusion of remifentanil and propofol (Figure 4C). A decrease in qCON (Figure 4A) took place as a consequence of the effect of the drugs, resulting in the transition from the awake state to anesthesia around t = 700s. Different events can be observed, steady state anesthesia (Figure 4A) begins right after the drug concentrations of propofol and remifentanil are lowered and stabilized at t = 1000s and lasts for 1000s (Figure 4C). Immediately afterwards, the remifentanil effect site concentration was increased, originating the new clinical event that seems to be followed by EEGδ(t), EEGθ(t), EEGα(t), EEGß(t) energies (Figure 4B), HR (Figure 4D) and δmax (Figure 4E).

The mean and standard deviation values of REG geometric features calculated on each anesthesia state are depicted in Table 2. Statistical significance level obtained comparing each two consecutive states (Awake vs LOC, LOC vs Anes, Anes vs BSR and BSR vs ROC) are also indicated. Values of REG Range, δmax, δrange, CBVrel and CBFest were higher at LOC state and minima at Anes, *p*-value < 0.005. Regarding the systolic area (AreaSyst), its value increased from BSR to ROC state, *p*-value = 0.006. No statistical differences were presented between the remainder consecutive states.

**TABLE 2:** Averaged values of the rheoencephalography REG(t) signal features, electroencephalogram EEG(t) features, and the clinical variables such as heart rate and mean arterial pressure, recorded during general anesthesia. The differences between consecutive anesthetic states (Awake vs LOC, LOC vs Anes, Anes vs BSR, BSR vs ROC) are indicated by means of the statistical significance level.

Index	Awake	LOC	Anes	BSR	ROC	Awake	LOC	Anes	BSR
						LOC	Anes	BSR	ROC
Cerebral hemodynamic features							
CBF lin							
Range	0.103 ± 0.061	0.130 ± 0.094	0.062 ± 0.025	0.071 ± 0.031	0.082 ± 0.049	n.s	***	n.s	n.s
Δtmax	379.2 ± 222.2	361.4 ± 154.4	277.0 ± 93.3	272.9 ± 45.8	357.5 ± 179.9	n.s	•	n.s	n.s
Δtmin	383.0 ± 227.0	352.1 ± 150.2	275.4 ± 80.5	285.8 ± 69.4	359.0 ± 186.0	n.s	n.s	n.s	n.s
Δt_min-max_	182.7 ± 145.0	162.7 ± 107.1	197.4 ± 74.8	114.6 ± 36.3	179.5 ± 133.0	n.s	•	n.s	n.s
Slope(α)	9E-4 ± 6E-4	11E-4 ± 9E-4	8E-4 ± 3E-4	8E-4 ± 4E-4	7E-4 ± 4E-4	n.s	•	n.s	n.s
Area	417.6 ± 257.8	393.9 ± 184.4	287.8 ± 83.2	301.0 ± 72.0	387.6 ± 210.1	n.s	•	n.s	•
AreaSyst	198.7 ± 159.8	181.5 ± 123.3	112.2 ± 77.7	120.7 ± 37.6	194.8 ± 150.1	n.s	n.s	n.s	*
δmax	19E-4 ± 12E-4	23E-4 ± 17E-4	12E-4 ± 4E-4	13E-4 ± 6E-4	14E-4 ± 8E-4	n.s	***	n.s	n.s
δrange	30E-4 ± 20E-4	33E-4 ± 22E-4	18E-4 ± 6E-4	20E-4 ± 9E-4	22E-4 ± 12E-4	n.s	***	n.s	n.s
CBVrel	1.015 ± 0.669	1.128 ± 0.822	0.593 ± 0.194	0.660 ± 0.295	0.747 ± 0.412	n.s	***	n.s	n.s
CBFest	51.91 ± 40.34	48.76 ± 31.14	33.99 ± 12.42	37.51 ± 18.86	34.87 ± 19.90	n.s	**	n.s	n.s
CBF PP							
SD1	0.015 ± 0.009	0.015 ± 0.009	0.009 ± 0.003	0.010 ± 0.004	0.011 ± 0.006	n.s	***	n.s	n.s
SD2	0.045 ± 0.027	0.058 ± 0.043	0.027 ± 0.011	0.031 ± 0.014	0.036 ± 0.022	n.s	***	n.s	n.s
SDratio	0.339 ± 0.074	0.295 ± 0.074	0.345 ± 0.054	0.329 ± 0.053	0.316 ± 0.060	*	*	n.s	n.s
SDarea	9E-4 ± 11E-4	12E-4 ± 16E-4	3E-4 ± 2E-4	4E-4 ± 3E-4	5E-4 ± 6E-4	n.s	***	n.s	n.s
CCM	3E-5 ± 2E-5	3E-5 ± 3E-5	3E-5 ± 1E-5	3E-5 ± 1E-5	3E-5 ± 2E-5	*	n.s	n.s	n.s
R	0.789 ± 0.084	0.834 ± 0.073	0.785 ± 0.059	0.802 ± 0.056	0.814 ± 0.063	*	*	n.s	n.s
Global hemodynamics							
HR	69.8 ± 13.8	64.9 ± 10.3	61.4 ± 9.6	60.1 ± 10.2	66.1 ± 14.0	n.s	n.s	n.s	n.s
MAP	99.1 ± 12.6	89.2 ± 20.6	76.7 ± 15.4	76.2 ± 16.0	81.4 ± 16.3	•	*	n.s	n.s
EEG based-parameters							
qCON	95.9 ± 8.04	47.0 ± 13.6	40.1 ± 9.4	24.2 ± 8.7	71.1 ± 15.0	***	•	***	***
EEGδ	−0.153 ± 0.095	−0.173 ± 0.100	−0.238 ± 0.152	−0.207 ± 0.117	−0.595 ± 0.298	n.s	n.s	n.s	***
EEGθ	−3.374 ± 0.627	−3.616 ± 0.616	−3.561 ± 0.618	−3.459 ± 0.670	−2.943 ± 0.540	n.s	n.s	n.s	***
EEGα	−4.387 ± 0.714	−3.360 ± 0.801	−2.954 ± 0.771	−3.245 ± 0.584	−2.278 ± 0.849	***	n.s	n.s	***
EEGß	−5.250 ± 0.905	−4.411 ± 0.654	−4.368 ± 0.640	−4.320 ± 0.634	−2.951 ± 0.800	***	n.s	n.s	***

Significant levels (*p*-value): n.s not significant; • < 0.05; * < 0.01; ** < 0.005; *** < 0.0005.

Value of the descriptor is expressed by mean ± standard deviation.

Results concerning to Poincaré plot features of REG segments for *τ* = 20 samples are reported in Table 2. SD1 presented similar values, in average, in Awake vs LOC, decreasing during anesthesia (LOC, m ± std = 0.015 ± 0.009; Anes, m ± std = 0.009 ± 0.003; *p*-value = 0.00005) and slightly increasing for BSR and ROC, but without recovering the initial values at Awake and LOC. Descriptor SD2 had a similar performance, except for the transition between Awake and LOC, where SD1 showed similar values while SD2 increased. Since SDarea is proportional to the product of SD1 and SD2, it followed similar behavior described for those two features comparing LOC vs Anes (*p*-value = 0.00007). Regarding SDratio and R descriptors, they presented opposite trends, as expected, with similar variances for all patient states: SDratio showed an absolute minimum for LOC, while R had its maximum in this same state. Both descriptors were able to statistically differentiate Awake vs LOC (*p*-value = 0.0053 and *p*-value = 0.0065, respectively) and LOC vs Anes (*p*-value = 0.0076 and *p*-value = 0.00873, respectively), as it is indicated in Table 2. Finally, CCM presented a maximum for the Awake state and a minimum for LOC state (*p*-value = 0.0095), presenting similar values for all states except for LOC.

The trends of all extracted features present changes along the anesthetic procedure for *τ* = 20 samples, mainly during LOC. However, only some of those changes are statistically significant. All the features except CCM were able to detect changes in the transition between LOC and Anes states. Regarding the differences between the Awake and LOC states, SDratio, CCM and R provided statistically significant results while none of the features led to positive results for the transitions among other anesthesia states.

The evolution of the EEG energy, qCON, HR and MAP across the identified anesthesia states is presented in Table 2. It is observed that qCON index statistically differentiates (*p*-value < 0.01) Awake to LOC, Anes to BSR and BSR to ROC state transitions, showing decreasing values from Awake to BSR but increasing at the recovery of consciousness (ROC) state (qCON (m ± std): Awake, 95.9 ± 8.04; LOC, 47.0 ± 13.6; Anes, 40.1 ± 9.4; BSR, 24.2 ± 8,7; ROC, 71.1 ± 15.0). The MAP could statistically differentiate LOC vs Anes (MAP (m ± std): LOC, 89.2 ± 20.6; Anes, 76.7 ± 15.4; *p*-value = 0.008), while HR was not able to differentiate any transition. Regarding to EEG frequency bands, a similar trend was observed in EEGα and EEGß with high statistical differences when comparing Awake vs LOC and BSR vs ROC. Both EEGδ and EEGθ energies only statistically differentiated BSR vs ROC states.

### 3.3 Causality analysis at different anesthesia events

For every general anesthesia event (steady state anesthesia, propofol infusion, remifentanil infusion, atropine infusion, ephedrine infusion, Trendelenburg position and passive leg raising) all couplings between pairs of variables (CBF lin, CBF PP, HR, MAP, EEG based-parameters, CePropo, CeRemi) are presented through Granger causality, with the aim of studying the causality between different physiological systems. For this reason, causalities among pairs of REG features are not considered as well as the causal links between pairs of EEG-based parameters.

#### 3.3.1 Steady state anesthesia

**FIGURE 5:**
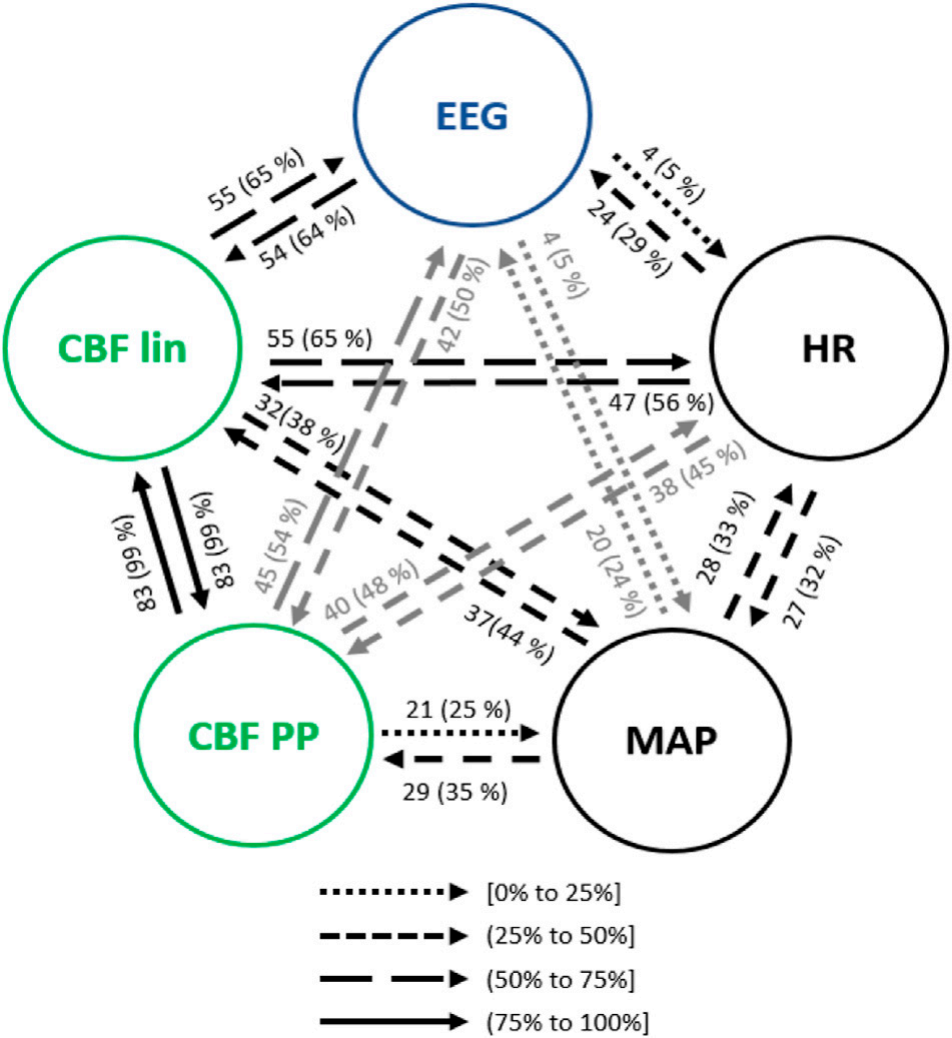
Main interactions between global hemodynamics (HR and MAP), EEG based-parameters, REG geometric features (CBF lin) and REG Poincaré plot (CBF PP) parameters during steady state anesthesia. The post hoc non-parametric U Mann-Whitney test and statistical significance level *p*-value < 0.005 were considered.

The main interactions between the physiological variables (HR, MAP, EEG based-parameters, CBF lin and CBF PP features) during steady state anesthesia are presented in Figure 5. Up to 99% of the analyzed patients showed a bilateral causal relationship between CBF lin and CBF PP parameters, since both sets of variables come from the same time series. Regarding the interactions between HR and CBF features, causalities from CBF to HR were more frequent than in the opposite direction, with the linear CBF (CBF lin) features showing a stronger role over the nonlinear ones (CBF PP). This relevance of the linear features is preserved in the causality study from and to MAP, even though in this case the causalities from MAP to the CBF features are more frequent than the opposite ones.

Both global (HR and MAP) and cerebral hemodynamics (CBF lin and CBF PP) presented causal relationships with EEG activity. EEG based-parameters had a similar occurrence of causality (5% of patients) towards HR and MAP, while HR presented a higher rate of causality towards EEG (29% of patients) than MAP (24% of patients). Regarding cerebral hemodynamics, the most relevant results rely on the 65% of causality from the CBF lin features to the EEG variables, which is one of the highest occurrences of interactions of the full system considered and therefore strongly suggests a modulation of EEG activity as a result of changes in the REG signals represented by their linear features. The Poincaré extracted features (CBF PP) showed a lower occurrence of causality (54% of patients) on EEG variables, but still higher than the ones provided by global hemodynamics HR and MAP of 29% and 24% of patients, respectively. Finally, the causality from EEG to CBF features was also higher for the linear features (CBF lin) when compared to the nonlinear parameters (CBF PP) extracted from REG signals, with 64% and 50% of patients, respectively.

No correlations were found between the causality indices and patient demographics and neither influences due to age, height, weight or BMI based on regression analysis.

#### 3.3.2 Propofol infusion event

The propofol effect site concentration, CePropo, was added to the analysis since it is not constant in general anesthesia scenario. However, it should only be considered as a causing variable, since it is collected from infusion pumps, resulting from the calculation of pharmacokinetic models and is not influenced by other physiological systems.

**FIGURE 6:**
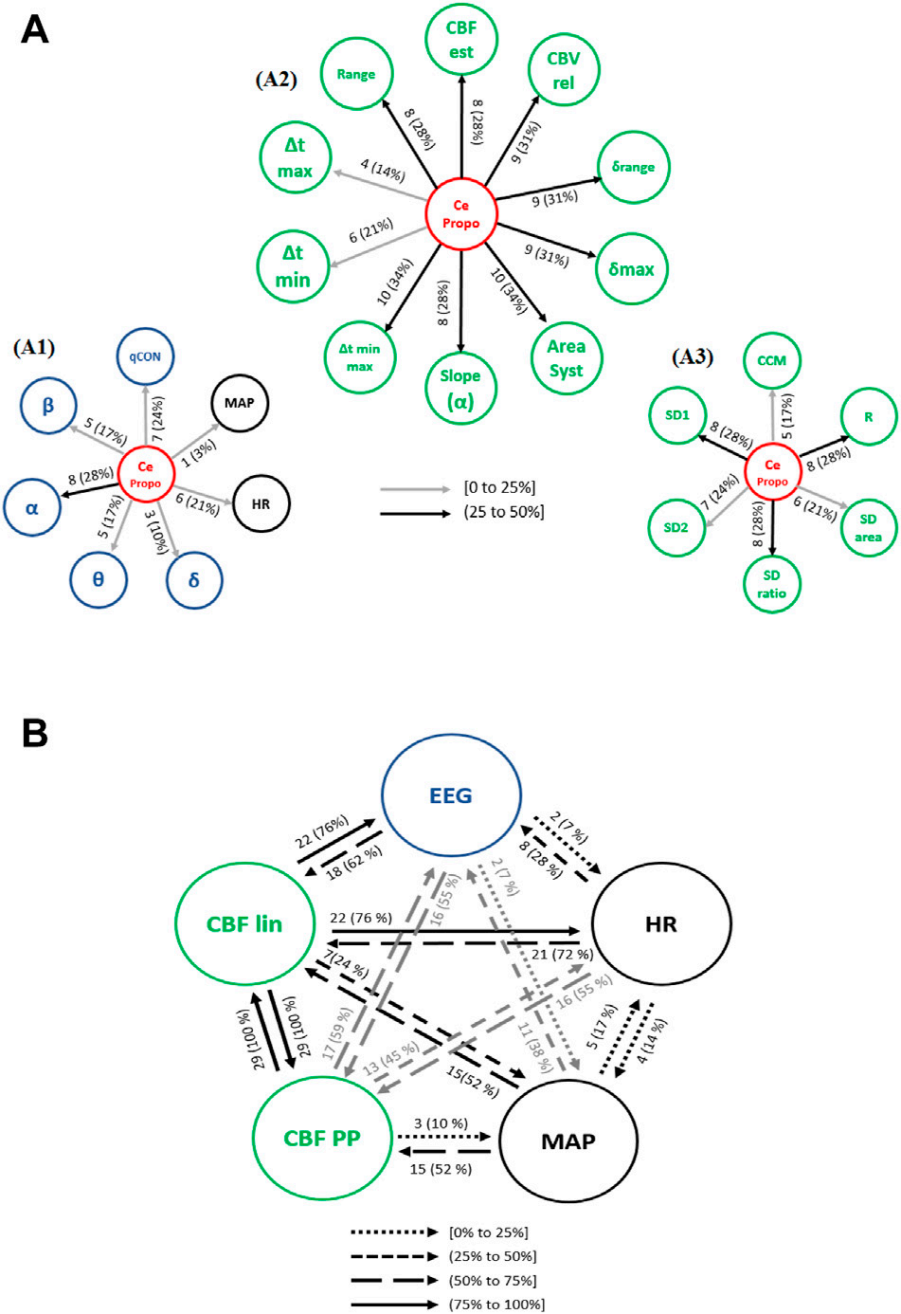
Causal interactions from **(A)** CePropo to: (A1) EEG based-parameters (δ, θ, α, ß) and global hemodynamics (HR, MAP), (A2) REG geometric features and (A3) REG Poincaré plot features. **(B)** Causal interactions between global hemodynamics, EEG based-parameters, REG geometric features (CBF lin) and REG Poincaré plot (CBF PP) parameters in propofol concentration. The post hoc non-parametric U Mann-Whitney test and statistical significance level *p*-value < 0.005 were considered.

The interactions from the propofol effect site concentration (CePropo) towards all the collected physiological data are represented in Figure 6A. Causality in the opposite direction was not assessed since it does not have any clinical interpretation as previously stated. Among all the EEG bands (Figure 6A1), CePropo has the highest interaction with α (28% of patients), with similar results for its causality towards the qCON index (24% of patients). This indicates that the changes in propofol dosages are mainly affecting the α band and therefore projected in the overall depth of anesthesia assessment represented by the qCON index. The influence of CePropo in HR was detected in 21% of the patients, while causal relationships with MAP were limited to one patient. Regarding the effects of CePropo in the linear features extracted from REG signals (Figure 6A2), the causal relationships with higher occurrence were those towards Δtmin-max and AreaSyst, identified in 34% of the patients, followed by a 31% occurrence of causalities towards CBVrel, δmax and δrange. The less frequent interactions took place from CePropo to Δtmax and Δtmin. The Poincaré plot features (Figure 6A3) showed smaller occurrences, the higher ones associated to SD1, SDratio and R with 28% of patients, suggesting that CePropo is affecting the short-term variability of REG signals rather than the long-term one.

Besides the direct effects of propofol concentration changes in all the physiological variables under study, the causal relationships among hemodynamics and EEG might also be affected by the administration of the hypnotic drug. Figure 6B shows an overview of the existing causal interactions between global hemodynamics (HR, MAP), cerebral hemodynamics (CBF lin and CBF PP) and EEG related variables. Even though the detected interactions are similar to those during steady state anesthesia, several differences can be appreciated. For instance, the occurrence of causal interactions from HR and MAP towards CBF PP, CBF lin and EEG are higher, suggesting that changes in HR caused by propofol are projected in CBF and EEG. Additionally, causal effects from CBF lin to HR (76% of patients) and EEG (76% of patients) are also more frequent under propofol infusion, while the interactions between MAP and HR have a lower occurrence. Overall, changing the propofol effect site concentration elicits a higher number of interactions from both cerebral and global hemodynamics towards EEG.

The causality indices and patient demographics showed statistically significant correlations during changes in propofol effect site concentration (Table 3). Age proved to be correlated to the causality indices computed from REG features towards MAP, with correlations obtained for the REG slope (α), δrange, CBFest, SDratio and R. Among those, the linear parameters (REG slope (α), δrange and CBFest) presented increasing relations for increasing ages, while for the Poincaré based features (SDratio and R) the opposite behavior was detected. Moreover, qCON towards the REG slope (α) positively correlated with age. The influence of patient’s height in the causality indices was only relevant for the causal links from SDratio and R to MAP, with taller patients related to higher values of the causality indices. In contrast, weight showed a more determinant role, patients with higher weight presented lower causality indices from CePropo to MAP, from Δtmin to MAP and from CBFest to the EEGα band. Nonetheless, the highest correlations were detected for the causality links from the EEGθ band to Δtmin and AreaSyst, with a positive correlation. Finally, BMI demonstrated to be relevant in the interactions between REG features and EEG. BMI was positively correlated with the causality relation form the EEGθ to Δtmin-max relation and AreaSyst, while it presented a negative correlation with the indices calculated from Δtmin-max and AreaSyst to EEGδ. Influence of BMI was found in these causality indices on the level of adjusted *R* ^2^ = 0.7. However, no influences in causality indices due to age, height or weight based on regression analysis could be found.

**TABLE 3:** Spearman correlation (ρ) between the causality indices and patient demographic.

From	To	Demographic	ρ
During changes of propofol effect site concentration			
CBFest	MAP	age↑	0.573
Slope (α)	MAP	age↑	0.523
δrange	MAP	age↑	0.548
qCON	Slope (α)	age↑	0.574
R	MAP	age↓	−0.535
SDratio	MAP	age↓	−0.553
R	MAP	height↑	0.543
SDratio	MAP	height↑	0.537
EEGθ	AreaSyst	weight↑	0.618
EEGθ	Δtmin	weight↑	0.621
CBFest	EEGα	weight↓	−0.537
Δtmin	MAP	weight↓	−0.577
EEGθ	AreaSyst	BMI↑	0.504
EEGθ	Δtmin-max	BMI↑	0.518
AreaSyst	EEGδ	BMI↓	−0.569
Δtmin-max	EEGδ	BMI↓	−0.524
During changes of remifentanil effect site concentration			
CCM	MAP	age↑	0.668
HR	EEGθ	weight↑	0.672
HR	SD1	weight↑	0.646
EEGß	δmax	weight↑	0.640
EEGα	Δtmax	weight↑	0.752
AreaSyst	HR	weight↓	−0.673
Δtmin-max	HR	weight↓	−0.707
CBVrel	qCON	weight↓	−0.698
EEGδ	R	weight↓	−0.639
EEGθ	Slope (α)	weight↓	−0.643
EEGß	δmax	BMI↑	0.637
EEGδ	R	BMI↓	−0.695
EEGδ	SDratio	BMI↓	−0.658

Increasing causality is denoted by ↑

Statistical significance *p*-value < 0.01.

#### 3.3.3 Remifentanil infusion event

Causalities from CeRemi towards other variables should be taken into account since, as discussed for CePropo, CeRemi data are the result of the calculation of pharmacokinetic models and are not affected in any way by other physiological data, only depend on patient demographics.

**FIGURE 7:**
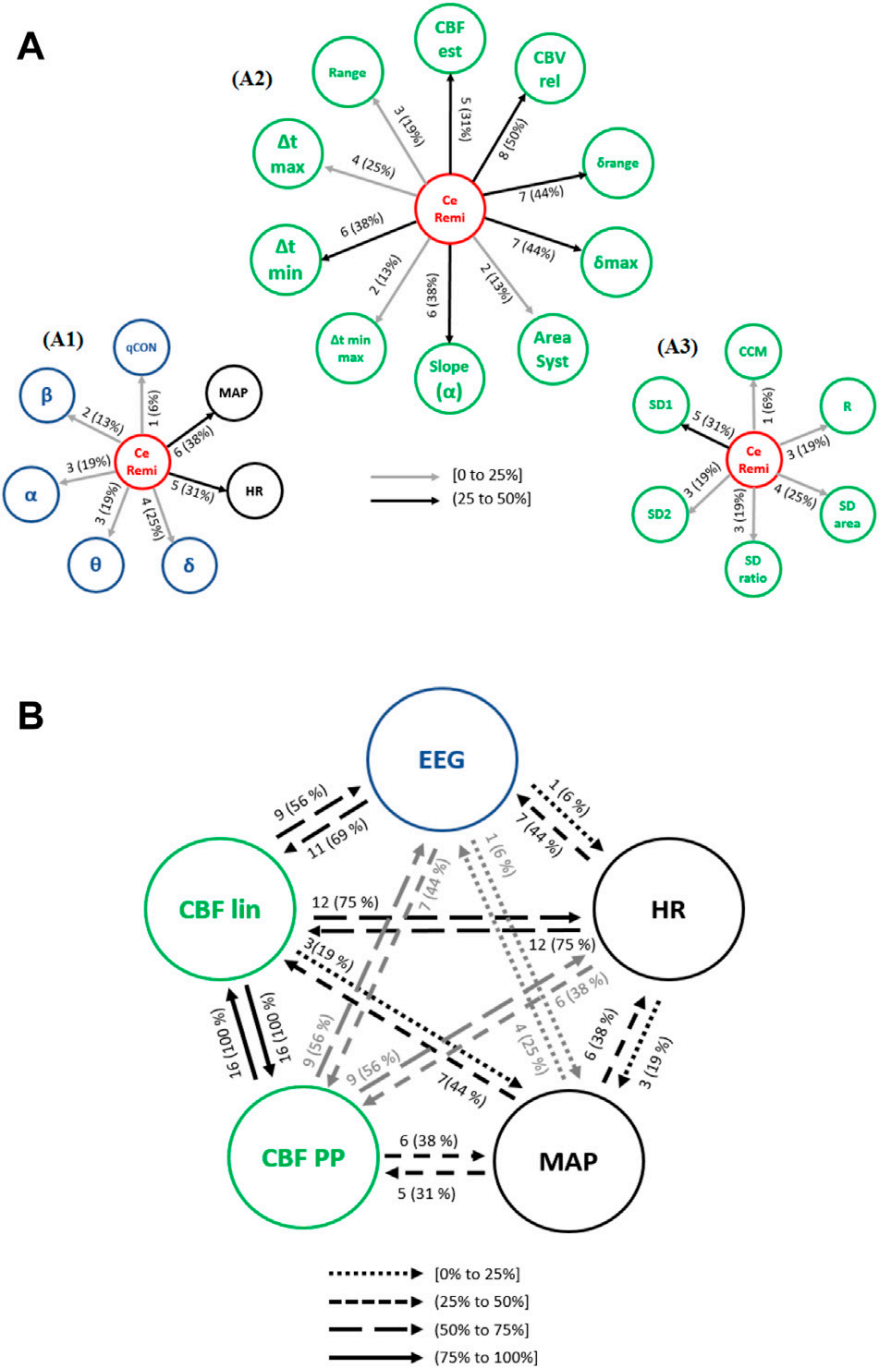
Causal interactions from **(A)** CeRemi to: (A1) EEG based-parameters (δ, θ, α, ß) and global hemodynamics (HR, MAP), (A2) REG geometric features and (A3) REG Poincaré plot features. **(B)** Causal interactions between global hemodynamics, EEG based-parameters, REG geometric features (CBF lin) and REG Poincaré plot (CBF PP) parameters in remifentanil concentration. The post hoc non-parametric U Mann-Whitney test and statistical significance level *p*-value < 0.005 were considered.

Causal interactions from CeRemi towards EEG based-parameters, global hemodynamics (HR and MAP), linear features (CBF lin) and nonlinear features (CBF PP) are depicted in Figure 7A. The effects of CeRemi on EEG variables (Figure 7A1) have occurrences up to 25%, almost inexistent towards the qCON index, but slightly higher for α, θ and δ bands. However, causal relationships between CeRemi and global hemodynamics represented by HR and MAP were more frequent, reaching an incidence of 31% and 38%, respectively. Regarding the causal effects of CeRemi towards the linear features of CBF (Figure 7A2), the highest occurrences took place in the causality from CeRemi to CBVrel (up to 50%), followed by δrange and δmax (44% of patients). The weakest causality was detected towards Δtmin-max and AreaSyst, and this is one of the main differences when comparing causal effects elicited by CePropo and CeRemi. Finally, for the REG Poincaré plot features, the most frequent interaction was from CeRemi to SD1 (31% of patients), as detected as well in the CePropo analysis, suggesting that changes in remifentanil infusion did also affect short-term variability of REG signals.

The occurrence of causal interactions between HR, MAP, EEG and CBF linear and nonlinear parameters is presented in Figure 7B. When compared to steady state anesthesia, the causal effects of HR on EEG and CBF lin features are enhanced, as well as the effects of CBF PP on EEG. On the contrary, causal relationships of CBF lin features on EEG have lower occurrence. Moreover, when comparing CeRemi changes to CePropo changes, causality from HR to EEG is much more frequent under CeRemi analysis (44% of patients), while causality from MAP to EEG decreases, allowing to consider that CePropo modulates EEG changes through MAP while CeRemi influences EEG by means of HR. With respect to other significant differences, it should also be mentioned that CBF linear and nonlinear features have less frequent causal links with EEG variables, when compared to the analysis of CePropo changes. This finding is consistent with the fact that CePropo is acting at a cerebral level, reducing brain metabolism, while CeRemi has a less pronounced influence in EEG signals.

The causality indices obtained for several pairs of variables were highly correlated with patient demographics as summarized in Table 3, during changes in remifentanil effect site concentration. Age presented a positive correlation with the causality indices from CCM towards MAP, hence indicating that older patients presented higher causality indices between those two physiological parameters. Nonetheless, patient weight was the demographic variable showing more correlation value in the causal interactions detected under remifentanil dosage changes. For instance, the causality indices from Δtmin-max and AreaSyst towards HR showed a negative correlation with weight, suggesting that causality from REG to HR is enhanced in patients with lower weight (regressive analysis with adjusted *R* ^2^ = 0.6). A negative correlation was also obtained for the causal link from CBVrel to qCON, from EEGθ band to the slope of REG, and from the EEGδ band to the Poincaré descriptor R, while positive correlations were found for the interactions from EEGα to Δtmax, from EEGβ to δmax, from HR to SD1 and from HR to the EEGθ band. Some of those results were replicated for the BMI analysis, namely the causality from EEGβ to δmax and from EEGδ to R. Additionally, BMI presented a negative correlation from EEGδ to SDratio. Those correlations suggest that links between general hemodynamics, EEG activity and REG features under changes of remifentanil dosage are sensitive to the main characteristics of the patients being monitored, with weight being the key factor that influences causality from REG to EEG and HR to REG features, with a regression relation with adjusted *R* ^2^ = 0.6.

#### 3.3.4 Atropine infusion event

**FIGURE 8:**
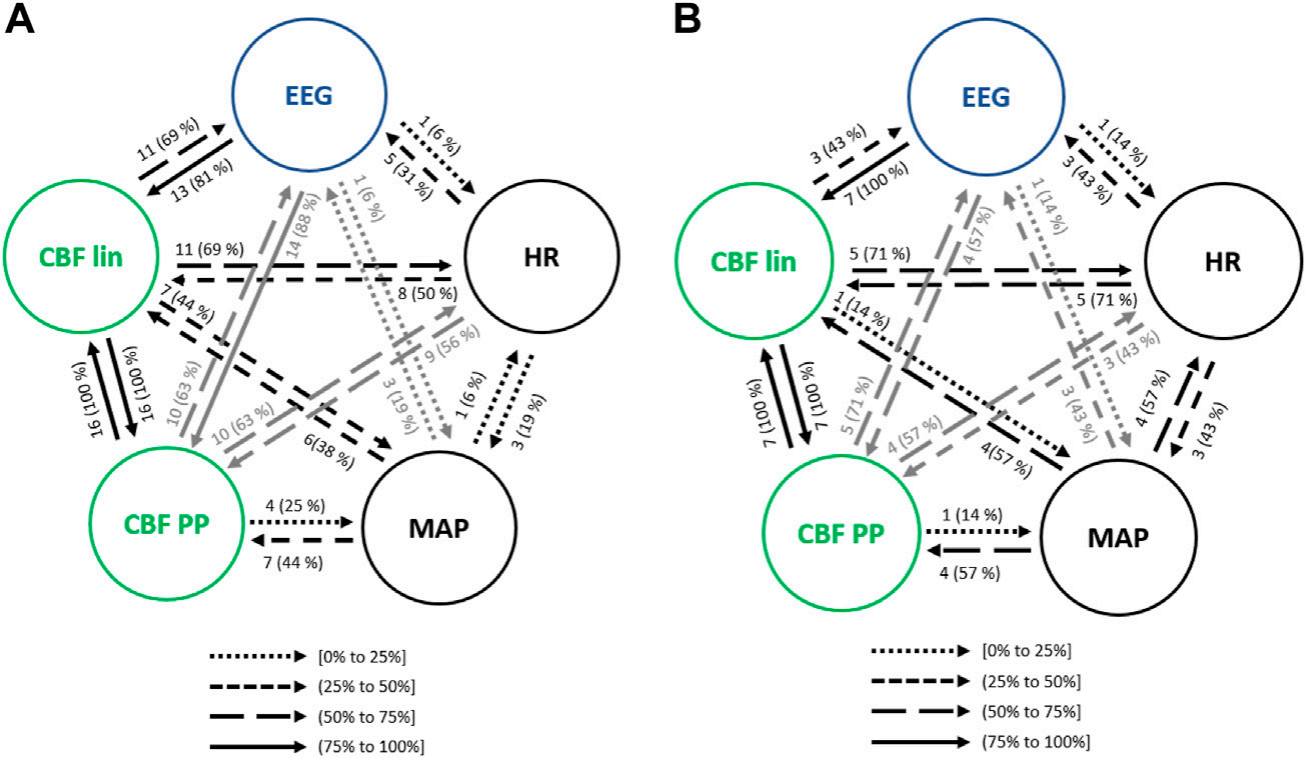
Causal interactions between global hemodynamics, EEG based-parameters, REG geometric features (CBF lin) and REG Poincaré plot (CBF PP) parameters **(A)** in atropine infusion and **(B)** in ephedrine infusion. The post hoc non-parametric U Mann-Whitney test and statistical significance level *p*-value < 0.005 were considered.

Figure 8A presents the interactions between EEG parameters, HR, MAP and CBF extracted features. Causalities emerging from HR were lower towards MAP and CBF lin when compared to steady state anesthesia, but higher towards CBF PP and EEG. Regarding MAP, the causal link towards CBF PP (44% of patients) showed a higher occurrence for atropine infusion, while all other links were detected with a lower frequency. Finally, the analysis of the interactions between EEG and REG features was enhanced during the administration of atropine (CBF lin with 81% and CBF PP with 88% of patients), suggesting that this drug affects the electrical brain activity.

Several correlations between the causality indices and the demographic data of the patients were identified as significant (Table 4). Age presented a negative correlation with the causality indices from qCON to δrange and from MAP to SD1, and a positive one from the REG features Δtmax, Δtmin and SD2 towards the EEGβ band. Furthermore, regression analysis indicated that older patients present causal links from REG to EEG (*R* ^2^ = 0.6). Several correlations between height and the analyzed set of causal links were also found to be significant. For instance, the causality from the EEGα band to Δtmax had a negative correlation with height, while positive correlations were obtained from EEGβ to δrange, from EEGθ to SD1, from θ to SDarea and from HR to EEGθ. Therefore, the links between EEG and CBF features during atropine infusion seem to be dependent on patient height (also with regression analysis adjustment of *R* ^2^ = 0.6). Finally, weight was positively correlated with the causality index computed from CBVrel to the EEGδ band, as well as BMI. Additionally, BMI showed positive correlations from δmax, CBFest and SDarea towards the EEGδ band, and from Δtmax towards EEGβ. Increased BMI is hence related to enhanced causality from REG features towards electrical brain activity, with a regression analysis with adjusted *R* ^2^ = 0.6.

**TABLE 4:** Spearman correlation (ρ) between the causality indices and patient demographic.

From	To	Demographic	ρ
During atropine infusion			
SD2	EEGß	age↑	0.694
Δtmax	EEGß	age↑	0.780
Δtmin	EEGß	age↑	0.689
MAP	SD1	age↓	−0.736
qCON	δrange	age↓	−0.638
HR	EEGθ	height↑	0.664
EEGθ	SD1	height↑	0.693
EEGθ	SDarea	height↑	0.664
EEGß	δrange	height↑	0.715
EEGα	Δtmax	height↓	−0.748
CBVrel	EEGδ	weight↑	0.679
CBFest	EEGδ	BMI↑	0.723
CBVrel	EEGδ	BMI↑	0.749
SDarea	EEGδ	BMI↑	0.688
δmax	EEGδ	BMI↑	0.798
Δtmax	EEGß	BMI↑	0.692
During ephedrine infusion			
CCM	EEGα	height↓	−0.906
EEGδ	Range	weight↓	−0.955
EEGδ	Slope (α)	weight↓	−0.901
EEGδ	Range	BMI↓	−0.955
EEGδ	Slope (α)	BMI↓	−0.901

Increasing causality is denoted by ↑

Statistical significance *p*-value < 0.01.

#### 3.3.5 Ephedrine infusion event

As with the previous clinical scenarios analyzed, the interactions between the main sets of physiological variables were analyzed to assess the relationship between hemodynamics and brain activity (Figure 8B). One of the most relevant changes when compared to steady state anesthesia was the occurrence of the EEG causality towards CBF parameters, as well as the one from CBF lin to EEG (43% of patients) and all causal links emerging from HR and MAP, suggesting that the cardiovascular effects of ephedrine are also projected in brain activity. Some of those effects were also detected during the infusion of another vasoactive drug, atropine, even though in that case the causalities emerging from MAP and HR were in general lower, while those from EEG to CBF PP and from CBF lin to both MAP and EEG were enhanced.

Table 4 reflects the correlations between the causality indices and patient demographics. Age was not a relevant factor during ephedrine infusion. Decreasing height is correlated to increased causality between CCM and the EEGα band and also it is detected an influence to these causality indices from a regression with adjusted *R* ^2^ = 0.8. The highest correlation was detected between weight and the causality index from the EEGδ band towards the REG slope (*ρ* = −0.955, *R* ^2^ = 0.783), followed by the one from the EEGδ band towards the REG range (*ρ* = −0.901, *R* ^2^ = 0.804). Both correlations were also detected for BMI, suggesting that lower weight and BMI are associated to higher causality from EEG towards CBF features.

#### 3.3.6 Trendelenburg positioning

**FIGURE 9:**
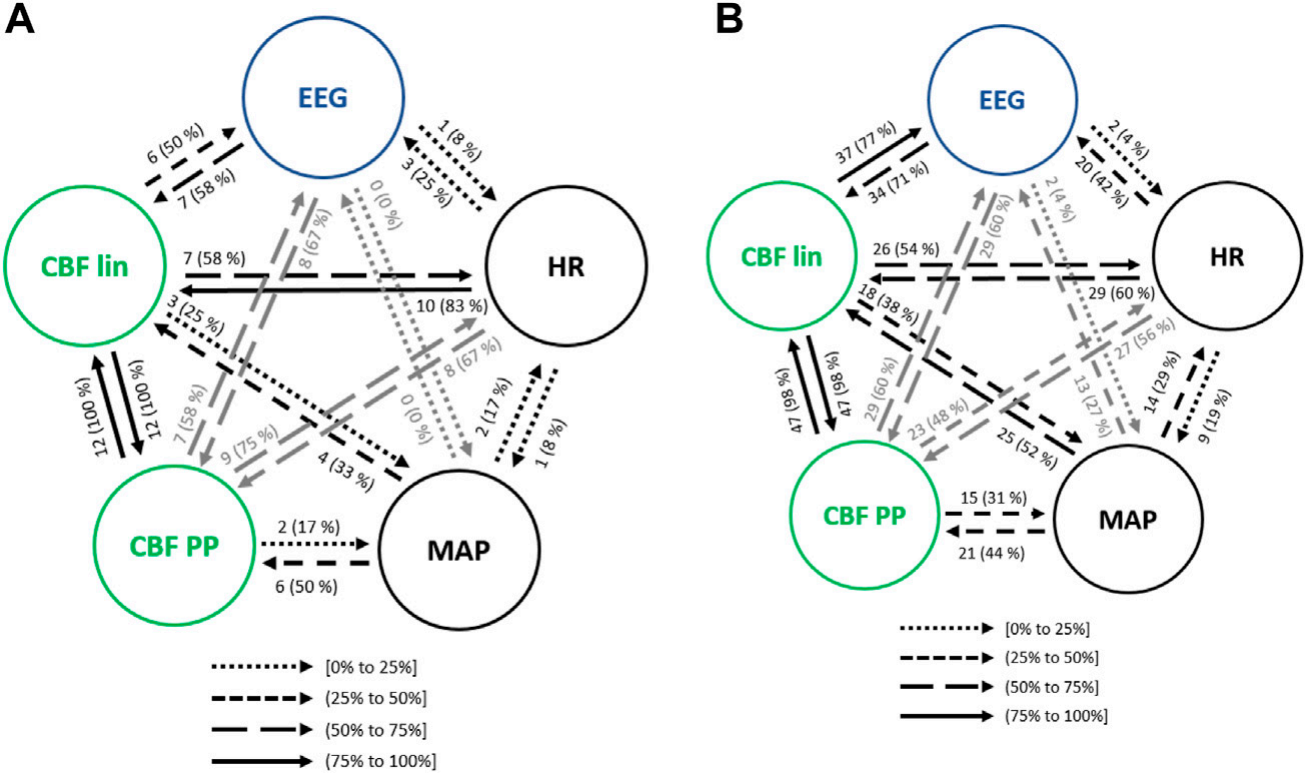
Causal interactions between global hemodynamics, EEG based-parameters, REG geometric features (CBF lin) and REG Poincaré plot (CBF PP) parameters **(A)** during Trendelenburg positioning and **(B)** during passive leg raising. The post hoc non-parametric U Mann-Whitney test and statistical significance level *p*-value < 0.005 were considered.

The transition of anesthetized patients from a supine position to Trendelenburg was assessed for causality. Considering the interactions between hemodynamics and brain activity signals (Figure 9A), HR showed less influence in MAP when compared to steady state anesthesia, but higher causal effects on CBF features, up to 83% for the linear ones. On the contrary, MAP caused lower interactions than in steady state, except for CBF PP, which were significantly higher. Moreover, while causal links between EEG and CBF PP were enhanced during Trendelenburg positioning when compared to stable anesthesia, links between EEG and CBF lin features showed lower occurrence.

Regarding the influence of demographic characteristics of the patients in the causal relationships previously analyzed (Table 5), age showed a high negative correlation with the causality indices from HR to AreaSyst and Δtmin-max, indicating that the younger the patients the higher the causality from HR towards REG features (with regression adjusted *R* ^2^ = 0.8). The causality index from the depth of anesthesia index, qCON, towards Δtmin-max and AreaSyst was negatively correlated with height, as well as the causality index from CCM to the EEGα band and from HR to EEGβ (with a regression analysis with adjusted *R* ^2^ = 0.5), suggesting that taller patients presented weaker causal links among those pairs of variables. The role of weight was limited to two statistically significant correlations: one from δmax to qCON, presenting higher causality in patients with less weight, and a second one from MAP to SD2, in which taller patients had higher causality index associated (with a regression analysis with adjusted *R* ^2^ = 0.5). Finally, lower BMI was associated to an enhanced causality from several REG features (δmax, δrange and CBVrel) to the qCON index while higher BMI resulted in a stronger causality (with regression analysis with adjusted *R* ^2^ = 0.8) from EEGδ to Range, from MAP to SD2 and from SD1 to EEGθ.

**TABLE 5:** Spearman correlation (ρ) between the causality indices and patient demographic during Trendelenburg positioning.

From	To	Demographic	ρ
HR	AreaSyst	age↓	−0.894
HR	Δtmin-max	age↓	−0.866
CCM	EEGα	height↓	−0.732
qCON	AreaSyst	height↓	−0.789
HR	EEGß	height↓	−0.732
qCON	Δtmin-max	height↓	−0.789
MAP	SD2	weight↑	0.872
δmax	qCON	weight↓	−0.746
SD1	EEGθ	BMI↑	0.769
EEGδ	Range	BMI↑	0.734
MAP	SD2	BMI↑	0.782
CBVrel	qCON	BMI↓	−0.769
δmax	qCON	BMI↓	−0.825
δrange	qCON	BMI↓	−0.769

Increasing causality is denoted by ↑

Statistical significance *p*-value < 0.01.

#### 3.3.7 Passive leg raise

Interactions among the physiological systems under study is presented in Figure 9B. Besides the bidirectional link between linear and nonlinear CBF features, the most frequent causality during passive leg raising takes place from CBF linear parameters towards EEG (77% of patients), suggesting that changes in cerebral hemodynamics are projected in brain activity. When compared to steady state anesthesia, higher causalities are detected, mainly from HR to CBF PP and EEG, from MAP to CBF features and, bilaterally, between CBF features and EEG. Additionally, causality from EEG to CBF PP is increased during patient positioning.

Since both Trendelenburg and passive leg raise provoke hemodynamic changes, it is worth comparing the causal interactions between both situations. Causalities emerging from MAP have higher occurrence under passive leg raising, as well as the interactions from CBF features to brain activity variables, and from EEG to CBF lin. However, causality from EEG to CBF PP is decreased, as well as from HR to CBF parameters. Furthermore, no statistically significant correlations were found between the causality indices and patient demographics, suggesting that the detected interactions were not dependent on patient characteristics.

## 4 Discussion and conclusions

The geometric features extracted from REG waves collected during general anesthesia have provided statistically significant results. Several geometric features were able to detect differences between LOC and Anes states: Range, δmax, δrange, CBVrel and CBFest. The evolution of those values suggests a generally decreased CBF and instantaneous blood flow velocity during anesthesia, as previously reported by (Conti et al., 2006; Fodale et al., 2007). CBFest and CBVrel decreased during general anesthesia. This phenomenon has been related with the vasoconstriction associated to the propofol administration (Rasmussen et al., 2010). It should be noted that values for those two features are not recovered after extubation. This is probably caused by the effects of propofol in hemodynamics, since at the time of extubation it has not been eliminated from the body (Morgan et al., 1990). The reduction of CBF and related parameters in the anesthetic state might seem inconsistent with the slight increases detected during BSR. Intuitively, the lower the anesthetic depth, the lower the brain metabolism is, CBV and CBF. However, it has been proved in rats that hemodynamic fluctuations at the brain level occur during general anesthesia: cortical electrical activity is accompanied by oscillations in cerebral hemodynamics (Liu et al., 2010). This might explain the small and non-significant increase of the CBF related parameters during BSR.

The Poincaré plot features were computed for a range of *τ* values from 1 to 20 samples, after the preprocessing stage of the general anesthesia dataset. Statistical differences were found between Awake-LOC and LOC-Anes transitions, with a wide range of parameters showing statistically significant differences: SDratio, CCM and R in the Awake-LOC transition and all features but CCM in the LOC-Anes transition, however CCM presented statistical differences between Anes and BSR. Within the 1 to 20 samples interval of *τ* tested, the upper values concentrated the highest amount of statistically significant differences among anesthetic states. It is therefore stated that a value of *τ* = 20 samples (0.08 s) is the most appropriate one for the analysis of REG signals during anesthesia. The performance of those parameters is dependent on the time lag *τ* used to reconstruct the signal attractor. Considering all results previously discussed from the information extracted of the REG signal features under anesthesia, REG analysis might be able to reflect CBF changes in REG waves. Concerning to global hemodynamics and EEG related parameters, qCON index differentiated across the different anesthesia states, but MAP just from LOC to Anes (*p*-value<0.01). Related to energies on the frontal EEG frequency bands, statistical differences were found between Awake vs LOC and BSR vs ROC. Similar results were found by (Sanjari et al., 2021) that applied transfer entropy on EEG signal for depth of anesthesia estimation, obtaining statistical differences between awake vs unconscious and unconscious vs. recovery EEG frequency bands.

Causal interaction analysis was applied to interpret how EEG, general hemodynamics and CBF evolve during general anesthesia under propofol and remifentanil. These interactions have been studied during steady state anesthesia, as well as during certain events occurring during surgery, such as anesthetic concentration changes, the administration of vasoactive drugs and patient positioning.

Even though literature on causal interactions involving REG signals is not available, several studies on brain activity and general hemodynamics describing heart-brain interactions have been published during both natural sleep and anesthesia. For instance (Faes et al., 2014), analyzed causal relationships among HRV and EEG during a full night sleep of subjects, observing a strong link between nonlinear beat-to-beat analysis and the power spectrum of the EEGδ band. Analogously, this EEGδ band was the one found in this study having a more frequent coupling with CBF measurements. Moreover, other studies do also support this link between hemodynamics and brain networks (Jurysta et al., 2003; Jurysta et al., 2006).

A brain-heart causality study during propofol anesthesia was published by (Won et al., 2019), concluding that causalities increased with depth of anesthesia and were stronger in the brain-heart direction than from the heart to the brain. Results obtained for the analysis herein presented suggest in fact that the most frequent interactions took place from cerebral hemodynamics to the EEG spectral densities (rather than in the opposite direction), that HR and MAP had closed loop relationships with cerebral hemodynamics and the depth of anesthesia index presented bilateral causal links with cerebral hemodynamics. Even though a larger physiological system was considered in this work, the obtained results are not consistent with those presented by (Won et al., 2019). Some differences exist in the study design, mainly based on the lowest age of the patients enrolled in Won’s study, with a majority of males and receiving midazolam drug. Further data should be collected under the same circumstances to figure out the root cause of the differences between both studies, since patient demographics have shown to play an important role both in the occurrence of causality and its strength.

Overall, the analysis of causal interactions during steady state anesthesia showed that hemodynamics and EEG activity are closely linked, often under closed loop interactions, and even though there is no consensus on the direction and strength of those links, their existence has been published by several research groups and has turned neurocardiology into a relevant topic under analysis (Chen et al., 2017; Scherbakov and Doehner, 2018). Besides the study of causal interactions among heart and brain hemodynamics and EEG activity during stable anesthesia periods, changes in the concentration of propofol were also analyzed to assess its influence in these causal links. The effects of propofol on hemodynamics are well-known, characterized by a MAP and HR depression, cerebral vasoconstriction and reduced CBF while preserving autoregulation (Dagal and Lam. 2009). However, until now no information exists regarding the causality between hemodynamics and EEG during its infusion. The study performed in our work reveals that during a change in propofol dosage, the number of interactions between hemodynamics and brain activity increases. Changes in HR and MAP provoked changes in CBF and EEG, with CBF linear and nonlinear features causing EEG modulation. Moreover, one of the strongest links was found between the propofol effect site concentration and the EEGα band, which is consistent with the fact that propofol provokes a shift of the EEG energy towards this band (Schwilden et al., 1989). Additionally, a causal link between the propofol concentration and the qCON index was detected, as expected, since changes in hypnotic dosages should translate into changes in depth of anesthesia.

Several propofol pharmacokinetic-pharmacodynamic models are used in routine clinical practice for induction and maintenance of propofol anesthesia. The parameters used in those models are exclusively based on patient demographics but do not take into account hemodynamics. Given the causal relationships between brain activity and hemodynamics, the inclusion of HR, MAP or CBF data in the models would probably make them more patient-specific and improve their accuracies. Several studies have been published on this topic. Sahinovic et al. (Sahinovic et al., 2017) raised a concern on the use of propofol models in patients with brain tumours, since those might alter propofol kinetics and dynamics and loose accuracy. Furthermore, a new set of models called Physiologically-Based PK Models (PBPK) have been developed to account for the effects of hemodynamics in the currently used compartments models (Jones and Rowland, 2013), since hemodynamic variables such as cardiac output have shown to be determinant for predicting the effects of propofol infusion (Adachi et al., 2013). The results for propofol infusion in this work support the hypothesis that those new models should be key for anesthesia personalization, thus enhancing the accuracy of target-controlled infusion (TCI).

Similar conclusions can be drawn from the analysis of remifentanil concentration changes. The use of remifentanil is associated to depressed hemodynamics, preserving cerebral autoregulation but lowering CBF (Dagal and Lam. 2009), and its administration together with propofol is known to produce some synergies in the modulation of the EEG waves and resulting depth of anesthesia index (Copot et al., 2015). The causal interactions between CeRemi and EEG related parameters revealed that the highest causality took place from CeRemi to EEGδ frequency band, followed by EEGα, EEGθ and EEGβ, but was almost inexistent with the qCON index. Those results are consistent with the EEG spectral analysis under remifentanil infusion published by Kortelainen et al. (Kortelainen et al., 2009), that highlighted the influence of remifentanil in the EEG spectrogram rather than to limit its effects to synergies with propofol. Hence, remifentanil modified the spectral content of the EEG of the patients under study while the qCON index remained unaffected. The causal relationships detected during CeRemi changes suggest that its causal effects in EEG, either directly or through hemodynamics, are less pronounced than those obtained for propofol, which is consistent with the fact that propofol is a hypnotic drug while remifentanil is an analgesic. Moreover, HR seems to be the link between CeRemi infusion and brain activity, while MAP played a more relevant role in propofol infusion. Together with the effects of propofol and remifentanil in the EEG activity, the causal relationships induced by vasoactive drugs such as atropine and ephedrine were also studied in order to find out to which extent those drugs could affect brain activity and depth of anesthesia. Both drugs are often administered during anesthesia to compensate bradycardia and/or hypotension provoked by hypnotics and analgesics, and are therefore producing HR and MAP increases to achieve hemodynamic stability.

Furthermore, the presented results provided information supporting the hypothesis that effects of atropine and ephedrine in EEG activity take place through the causal links between MAP, HR and CBF features towards EEG parameters, and vice versa. In a recently published case study (Jo et al., 2018), atropine was administered to a patient presenting very low depth of anesthesia values, including EEG suppression and a bradycardia episode. After the atropine infusion, hemodynamic stability was recovered together with recommended depth of anesthesia values. The authors related this episode to cerebral hypoperfusion, therefore suggesting that causal interactions exist between hemodynamics and brain activity, and that those are modulated through CBF.

Patient positioning was also considered as a potential factor influencing causal relationships between hemodynamics and EEG activity. Two different positions were assessed besides the standard supine position in steady state anesthesia: Trendelenburg and passive leg raising. Both positioning strategies are known to provoke changes in general hemodynamics, mainly in MAP (Fakhari et al., 2018), but information on their influence in EEG is scarce. Mallick et al. reported the dependence between the depth of anesthesia index and the steepness of the Trendelenburg position, establishing a relationship between both variables (Mallick et al., 2015). Considering the causal occurrences calculated in this work, HR and MAP do not seem to modulate directly EEG changes, but through alterations in CBF features that are further projected into EEG activity.

As part of the causality analysis, the role of patient demographics was assessed through correlation and hypothesis testing. Patient characteristics such as age, height, weight and BMI should be taken into account since those might enhance or prevent the existence of causal relationships and the intensity of the existing causal effects. For instance, during steady state anesthesia, lower ages were associated to a higher occurrence of causal links from CBF to EEG, as well as lower weight and BMI. However, the size of the database under study impaired a more detailed analysis of patient demographics in heart-brain links during anesthesia, being one of the limitations of this study. Other limitations that should be noted are the low number of recordings for some of the analyzed events, such as atropine or ephedrine infusions, and the concomitant effects of different factors, as for instance patient positioning taking place before or after a drug dosage change or the administration of a vasoactive drug.

During the causality study, linear and nonlinear CBF features have been independently considered in order to assess their individual performance. Even though they showed a 100% of causal effects among them in the majority of events under test, they revealed different occurrences of causal relationships with brain activity and global hemodynamics. During propofol infusion, bilateral causality between linear CBF features and HR was much more frequent than between Poincaré plot features of REG signals and HR, while those showed similar values during steady state anesthesia. In contrast, during atropine infusion, effects of MAP on CBF parameters were more frequent towards the Poincaré features. The use of a larger dataset would allow to further compare the performance of both algorithms, but results herein presented suggest that they are closely related to each other but the integration of the information contained in both sets of features improves the assessment of causality.

As a conclusion, results from this study confirm the hypothesis that during general anesthesia causal interactions among global hemodynamics, cerebral hemodynamics and EEG neural activity take place. And, as a consequence, clinical decisions made to achieve hemodynamical stability have effects at a neural level, as well as changes in anesthetic dosages would interfere both in global and brain hemodynamics. REG signals provided an assessment of brain hemodynamics, with both linear and nonlinear features contributing to the heart-brain interactions, revealing its potential as a monitoring tool for anesthesia management. Finally, CBF estimators demonstrated to contain information allowing to understand the coupling between hemodynamics and neural activity, and should therefore be integrated in routine clinical care, mainly in patients in which causal relationships might be impaired or altered due to pathological or intrinsic conditions.

## Data Availability

The data analyzed in this study is subject to the following licenses/restrictions: The authors have not included the de-identified set of the data used in this work because of data restriction policies imposed by the Ethical Committee. Requests to access these datasets should be directed to cgz@quantiummedical.com.
